# Germline mutations in Japanese familial pancreatic cancer patients

**DOI:** 10.18632/oncotarget.12490

**Published:** 2016-10-06

**Authors:** Erina Takai, Shinichi Yachida, Kyoko Shimizu, Junji Furuse, Emi Kubo, Akihiro Ohmoto, Masami Suzuki, Ralph H. Hruban, Takuji Okusaka, Chigusa Morizane, Toru Furukawa

**Affiliations:** ^1^ Division of Cancer Genomics, National Cancer Center Research Institute, Tokyo, Japan; ^2^ Department of Gastroenterology, Institute of Gastroenterology, Tokyo Women's Medical University, Tokyo, Japan; ^3^ Department of Medical Oncology, Kyorin University School of Medicine, Mitaka, Japan; ^4^ Department of Hepatobiliary and Pancreatic Oncology, National Cancer Center Hospital, Tokyo, Japan; ^5^ Department of Pathology and Oncology, The Sol Goldman Pancreatic Cancer Research Center, The Johns Hopkins University School of Medicine, Baltimore, MD, USA; ^6^ Institute for Integrated Medical Sciences, Tokyo Women's Medical University, Tokyo, Japan

**Keywords:** pancreatic cancer, familial predisposition, BRCA2, ATM, PALB2

## Abstract

Clinicopathologic and genetic features of familial pancreatic cancer (FPC) in Asian countries remain largely unknown. The main purpose of this study was to determine the prevalence of FPC and to define causative FPC-predisposition genes in a Japanese cohort with pancreatic ductal adenocarcinoma (PDAC). We reviewed 1,197 patients with a pathologically proven PDAC and found that 88 (7.3%) were FPC patients who had at least one first-degree relative with PDAC. There were no significant differences between the FPC cases and sporadic cases in terms of gender, age, tumor location, stage, family history of any cancer except PDAC, and personal history of smoking, other cancers, diabetes mellitus and chronic pancreatitis. In the FPC patients, we then investigated the prevalence of germline mutations in 21 genes associated with hereditary predispositions for pancreatic, breast and ovarian cancers by means of the next-generation sequencing using a custom multiple-gene panel. We found that eight (14.5%) of the 54 FPC patients with available germline DNA carried deleterious mutations in *BRCA2*, *PALB2*, *ATM*, or *MLH1*. These results indicate that a significant fraction of patients with PDAC in Japan have a family history of pancreatic cancer, and some of them harbor deleterious causative mutations in known FPC predisposition genes.

## INTRODUCTION

Pancreatic ductal adenocarcinoma (PDAC) is a devastating diagnosis for patients and their families [1]. In Japan, the incidence and mortality have shown an increasing trend over the past decades [2] and incidence and mortality rates are approximately twice of those in the United States [3]. To date, no efficient and affordable screening tests for PDAC are available [4].

A generally accepted definition of familial pancreatic cancer (FPC) is presence of at least a pair of affected first-degree relatives (FDRs: sibling–sibling or parent-child) in the family [5]. It is estimated that 5-10% of patients with PDAC is familial in the United States [6] and individuals with a family history of PDAC have been shown to have a greatly elevated risk of developing PDAC themselves [5]. A number of genes, including *BRCA1*, *BRCA2* [7], *PALB2* [8], and *ATM* [9], have been associated with-FPC in Western countries. The prevalence of deleterious mutations in these genes varies significantly in different populations (e.g., Ashkenazi Jews have high rates of germline *BRCA2* mutations) [10].

Although there have been three studies reporting that a significant fraction of patients with PDAC have a family history of PDAC (10 out of 200, 5.0%; 40 out of 577, 6.9%; 28 out of 688, 4.1%) in the Japanese population [11–13], the prevalence of deleterious FPC susceptibility gene mutations in the Asian population is poorly described.

In the present study, we retrospectively reviewed 1,197 PDAC patients diagnosed at one of two hospitals to evaluate incidence of FPC and we performed targeted-sequencing of germline variants for previously reported FPC susceptibility genes in the Western countries in 54 patients with available DNA, who had a family history of PDAC, and 13 patients who had a personal history of breast or ovarian cancer.

## RESULTS

### Clinicopathologic features in PDAC patients with a family history of PDAC

A total of 955 patients were identified as having been diagnosed with PDAC between 2002 and 2013 in the National Cancer Center Hospital. Of these, 48 were excluded because of insufficient information, especially on their family histories, and eventually, 907 patients were enrolled, 68 (7.5%) of whom fulfilled the criterion of FPC in which at least a pair of FDRs had been diagnosed with PDAC. In addition, 290 patients were enrolled in the Tokyo Women's Medical University Hospital. Among them, 20 (6.9%) met criteria for FPC. Clinicopathologic features compared between FPC patients and PDAC patients without a family history of PDAC in FDRs are summarized in Table 1. There were no significant differences between groups in terms of gender, age, smoking history, tumor location, UICC stage, family history of any cancer except PDAC in FDRs, personal medical history of other cancer, diabetes mellitus and chronic pancreatitis in both hospitals.

**Table 1:** Comparison of clinicopathologic features between PDAC patients with and without a family history of PDAC in FDRs

Demographics	National Cancer Center Hospital (n = 907)			Tokyo Women's Medical University Hospital (n = 290)		
	A family history of PDAC in FDRs			A family history of PDAC in FDRs		
	Yes (n = 68)	No (n = 839)	*P* -value	Yes (n = 20)	No (n = 270)	*P* -value
**Gender**			0.0741			0.3327
Male	32 (47.1%)	491 (58.5%)		9 (47.4%)	163 (60.8%)	
Female	36 (52.9%)	348 (41.5%)		10 (52.6%)	105 (39.2%)	
Unknown	0	0		1	2	
**Age**						
Median, yrs	64	65		69	68	
Range, yrs	35-83	33-90		47-82	26-95	
**Smoking history**			0.4472			0.3229
Yes	30 (44.8%)	414 (50.0%)		10 (55.6%)	97 (41.6%)	
No	37 (55.2%)	414 (50.0%)		8 (44.4%)	137 (58.5%)	
Unknown	1	11		2	36	
**Tumor location**			0.0585			0.7480
Head	24 (35.3%)	416 (49.9%)		9 (45.0%)	138 (53.9%)	
Body/tail	42 (61.8%)	405 (48.6%)		9 (45.0%)	118 (46.1%)	
Whole	2 (2.9%)	13 (1.6%)		0	0	
Unknown	0	5		2	14	
**UICC stage**			0.3712			Unevaluable
0/IA	2 (2.9%)	23 (2.8%)		2 (10.0%)	7 (2.8%)	
IB	5 (7.4%)	38 (4.5%)		1 (5%)	1 (0.4%)	
IIA	4 (5.9%)	124 (14.8%)		0	20 (7.4%)	
IIB	9 (13.2%)	94 (11.2%)		0	5 (7.9%)	
III	15 (22.1%)	199 (23.8%)		6 (30.0%)	90 (35.4%)	
IV	33 (48.5%)	358 (42.8%)		9 (45.0%)	131 (51.6%)	
Unknown	0	3		0	16	
**Family history of any cancer in FDRs**			0.0762			0.4840
Yes	33 (52.3%)	473 (64.2%)		10 (50.0%)	110 (40.9%)	
No	30 (47.6%)	264 (35.8%)		10 (50.0%)	159 (59.1%)	
Unknown	5	102		0	1	
**Past medical history of other cancer**			0.3272			0.1052
Yes	7 (21.9%)	145 (30.7%)		6 (30.0%)	40 (14.9%)	
No	25 (78.1%)	327 (69.3%)		14 (70.0%)	229 (85.1%)	
Unknown	36	367		0	1	
**Diabetes mellitus**			0.4030			0.6238
Yes	20 (51.3%)	214 (43.5%)		5 (25.0%)	86 (31.9%)	
No	19 (48.7%)	278 (56.5%)		15 (75.0%)	184 (68.1%)	
Unknown	29	347		0	0	
**Chronic pancreatitis**			1.0000			1.0000
Yes	0	6 (2.9%)		0	3 (1.1%)	
No	11 (100%)	199 (97.1%)		20 (100%)	267 (98.9%)	
Unknown	57	634		0	0	

FDRs, first-degree relatives; PDAC, pancreatic ductal adenocarcioma; UICC, the Union for International Cancer Control.

### Analysis of germline variants

Among the 88 FPC patients we identified, germline DNA samples were available from 54 patients. We performed targeted deep sequencing using a massively parallel sequencer for 21 genes known to be associated with hereditary predispositions for pancreatic, breast, and ovarian cancers, namely, *ATM*, *BARD1*, *BRCA1*, *BRCA2*, *BRIP1*, *CDH1*, *CHEK2*, *MLH1*, *MRE11*, *MSH2*, *MSH6*, *MUTYH*, *NBN*, *PALB2*, *PMS1*, *PMS2*, *PTEN*, *RAD50*, *RAD51C*, *STK11*, and *TP53*. The unique sequence depth was 714.1× on average (range, 502.8× – 1126.1×). The FPC patients had 18 variations on average (range 9-28) in the 21 targeted genes. Eight (14.5%) of the 54 FPC patients carried deleterious variants, three in *BRCA2*, two in *PALB2*, two in *ATM*, and the remaining one in *MLH1*, as summarized in Table 2. These deleterious variants were verified successfully with Sanger sequencing. One of the mutations found in *BRCA2* gene, c.9076C>T/p.Gln3026Ter, has previously been identified in Japanese families with a history of breast or ovarian cancer [14, 15]. Details for clinicopathologic features in FPC patients with the deleterious mutations are shown in Table 3. All of tumors having developed in patients with these deleterious mutations were histopathologically conventional ductal adenocarcinomas. The youngest age of onset for PDAC in the 8 patients was 45 years old. The patient (ID, NCCH-16) who carried a nonsense mutation in *ATM* had a personal history of breast cancer and ureter cancer. We also classified six mutations into variants of unknown significance (VUS) (Supplementary Table S1).

**Table 2:** Deleterious mutations in 21 genes associated with hereditary predisposition for pancreatic, breast and ovarian cancers in PDAC patients with a family history of PDAC

	Patient ID	Gender	Gene	Sub region	Type of mutation	Nucleotide change	Amino acid change	1000_GenomeMAF (JPN)	ExAC Browser (East Asian)	HGVBSnpDB MAF	PROVEN (Score)	SIFT (Score)	Polyphen2 HDIV	Polyphen2HDIV_class	InSIGHT	LOVD IARC	ClinVar
1	NCCH-3	Male	*MLH1*	CDS12	Missense	c. 1153 C>T	p. Arg385Cys	NA	0.0003469	0.002061856	Deleterious (−7.06)	Damaging (0)	1.000	PROBABLY DAMAGING	class_4		Likely pathogenic
2	NCCH-5	Female	*ATM*	CDS49	Nonsense	c. 7456 C>T	p. Arg2486Ter	NA	NA	NA	NA	NA	NA				Pathogenic
3	NCCH-14	Female	*PALB2*	CDS4	Frameshift	c. 393_394 insC	p. Val32Argfs*3	NA	NA	NA	NA	NA	NA				NA
4	NCCH-16	Female	*ATM*	CDS6	Nonsense	c. 742 C>T	p. Arg248Ter	NA	NA	NA	NA	NA	NA				Pathogenic
5	NCCH-22	Female	*PALB2*	CDS4	Frameshift	c. 1195_1196 insACAGTGC	p. Pro399His fs*4	NA	NA	NA	NA	NA	NA				NA
6	NCCH-104	Female	*BRCA2*	CDS15	Frameshift	c. 7662_7663 insAA	p. Asn2556Lysfs*93	NA	NA	NA	NA	NA	NA			NA	Pathogenic
7	TWMU-1-1	Male	*BRCA2*	CDS10	Frameshift	c. 3571 delA	p. Lys1991Serfs*6	NA	NA	NA	NA	NA	NA			NA	NA
8	TWMU-7-1	Male	*BRCA2*	CDS2	Nonsense	c. 9076 C>T	p. Gln3026Ter	NA	NA	NA	NA	NA	NA			NA	Pathogenic

PDAC, pancreatic ductal adenocarcinoma

**Table 3:** Clinicopathologic data for each familial pancreatic cancer patient with a deleterious germline mutation

	Patient ID	Age	Gender	Gene with pathogenic mutation	UICC-stage	Smoking history (Brinkman index)	Tumor location	Family history of other cancer in FDRs	Past medical history of other cancer	Diabetes mellitus	Chronic pancreatitis
1	NCCH-3	62	Male	MLH1	IV	+ (630)	Body/tail	-	-	-	-
2	NCCH-5	55	Female	ATM	IV	-	Body/tail	Father (Gastric cancer, Colon cancer, Prostate cancer)	-	-	-
3	NCCH-14	45	Female	PALB2	IB	-	Body/tail	-	-	-	-
4	NCCH-16	67	Female	ATM	IIB	+ (510)	Head	-	Breast cancer, Ureter cancer	-	-
5	NCCH-22	58	Female	PALB2	IIB	-	Body/tail	Father (Brain tumor)	-	-	-
6	NCCH-104	52	Female	BRCA2	IB	-	Body/tail	-	-	-	-
7	TWMU-1-1	69	Male	BRCA2	III	-	Body/tail	-	-	-	-
8	TWMU-7-1	64	Male	BRCA2	IB	-	Head	-	-	+	-

### PDAC patients with a personal history of breast or ovarian cancer

PDAC patients with a history of breast and/or ovarian cancer may have a genetic predisposition common to FPC. Among the 907 PDAC patients in the National Cancer Center Hospital, 36 patients (4.0%) had a personal history of breast and/or ovarian cancer. Among 290 PDAC patients in the Tokyo Women's Medical University Hospital, 10 (3.4%) had a personal history of breast cancer. Among the 36 who had a personal history of breast and/or ovarian cancer in the National Cancer Center Hospital, germline DNA was available from 13 individuals. Among these 13 patients, 4 fulfilled the FPC criteria and their germline DNAs had been already examined. Then, we additionally examined mutations in the 21 genes in the remaining 9 patients without a family history of PDAC, therefore eventually, the germline mutation was investigated in 63 patients in total in this study. Three (23%) of the 13 PDAC patients with a personal history of breast or ovarian cancer had deleterious mutations, two *BRCA2* mutations and one *ATM* mutation, the latter of which was described in the previous section (NCCH-16) (Table 4). One of mutations found in *BRCA2* gene, c.6952C>T/p.Arg2318Ter, has previously been identified in Japanese having a strong family history of breast cancer [15, 16]. Although four of these 13 patients had a family history of PDAC, only one (NCCH-16) of three patients with deleterious mutations had such a family history.

**Table 4:** Pathogenic mutations in 21 genes associated with hereditary predisposition for pancreatic, breast and ovarian cancers in PDAC patients with a personal history of breast and/or ovarian cancer

	Patient ID	Family history of PDAC	Gender	Gene	Sub region	Type of mutation	Nucleotide change	Amino acid change	1000_GenomeMAF (JPN)	ExAC Browser (East Asian)	HGVBSnDB MAF	PROVEN	SIFT	Polyphen2 HDIV	Polyphen2HDIV_class	IARC class	ClinVar
1	NCCH-16	Yes	Female	*ATM*	CDS6	nonsense	c. 742 C>T	p. Arg248Ter	NA	0.0001156	NA	NA	NA	-	-		Pathogenic
2	NCCH-27	No	Female	*BRCA2*	CDS12	nonsense	c. 6952 C>T	p. Arg2318Ter	NA	NA	NA	NA	NA	-	-	5 - Definitely pathogenic	Pathogenic
3	NCCH-31	No	Female	*BRCA2*	Intron 16	splice	c. 7806-1 G>T	?	NA	NA	NA	NA	NA	-	-	5 - Definitely pathogenic	Pathogenic

PDAC, pancreatic ductal adenocarcinoma

## DISCUSSION

A better understanding of clinicopathologic and genomic features of FPC in each population should afford new opportunities for therapeutic intervention and clinical management of PDAC patients with a family history of PDAC. However, such features remain largely unexplored in Asian countries. Our present study provides compelling evidence that a subset of PDAC in the Japanese population is familial and attributable to germline mutations in known familial pancreatic cancer genes.

The prevalence of FPC found in the present study of a relatively large Japanese cohort from two hospitals is similar to that reported in the Western world [6]. Cho and colleague reported that 8 (7.2%) of 110 patients with PDAC met criteria for FPC in Korea [17]. Although the number of patients in the Korean study is small, the frequency of FPC is similar to our study. The age of onset of PDAC in some studies is reported to be slightly younger in FPC cases than in sporadic cases in the Western countries [18], though there was no significant difference in age in our Japanese population.

The heterogeneity of mutations uncovered in our Japanese cohort indicates the value of using a multiple-gene panel when evaluating patients with a family history of PDAC. Deleterious germline mutations in FPC cases were found in 14.5% of the Japanese cohort of FPC patients. Interestingly, these mutations were found to be heterogeneous but to primarily affect genes involved in DNA repair pathway, namely, *BRCA2*, *PALB2*, *ATM*, and *MLH1* [19]. *BRCA2* germline mutations are the most common in most Western cohorts of FPC [20, 21]. In the present study, 3.7% (2/54) of FPC patients had *PALB2* deleterious mutations. Jones et al. [8] identified *PALB2* mutations in 3 out of 96 American FPC families, which suggests that 3-4% of familial pancreatic cancer kindreds in Japan as well as USA likely to be attributable to germline *PALB2* mutations. Studies in other populations show varying mutation frequencies, ranging from absent in Dutch (0 out of 31) to 3.7% (3 out of 81) in Germans [22, 23].

Identification of germline mutations in *BRCA2*, *PALB2* and *ATM* may have valuable for implications of treatment. Cancers in which *BRCA2*, *PALB2*, or *ATM* have been biallelically inactivated are usually susceptible to poly(ADP-ribose) polymerase (PARP) inhibitors or platinum-based agents that facilitates double strand DNA breaks. This susceptibility could lead to an improved response and enhanced patient survival [24–26]. Consistent with this notion, Fogelman et al. [27] have recently shown that patients with a strong family history are more sensitive to platinum-based chemotherapy. Interestingly, PARP inhibition is currently being evaluated as a maintenance strategy after response to first-line platinum-based chemotherapy in patients with *BRCA*-related PDAC (https://clinicaltrials.gov/ct2/show/NCT02184195). In addition, our Japanese PDAC patients with a personal history of breast/ovarian cancer, although number is small, frequently carried deleterious mutations in these genes. This latter finding suggests that genetic testing is also valuable for treatment in such populations.

Following limitations could be considered in our study. First, our approach to classifying variants was conservative; several rare non-synonymous variants that were potentially deleterious were classified as VUS as noted in Supplementary Table S1, which could lead to underestimation of prevalence of deleterious mutation carriers. Second, the candidate target gene analysis approach employed in this study was intrinsically highly selective and, therefore, could lead to limited genetic results. Recently, Roberts et al. [28] conducted germline whole genome sequencing of 638 FPC patients and demonstrated that inherited PDAC-susceptible genes are highly heterogeneous, which has uncovered many genes, e.g., *BUB1B*, *FANCC*, and *FANCG*, in addition to the known classical causative FPC genes analyzed in this study. A Japanese large whole-exome sequencing project is ongoing to identify any characteristic or novel genes causative for FPC in Japan. Our approach would also miss some germline deletions. Finally, while we successfully identified germline alterations, we did not have tumor samples available to look for biallelic inactivation of these genes, and thus we were not able to definitively establish the functional loss of these genes.

The Japanese Familial Pancreatic Cancer Registry (JFPCR) was established in 2015 [29], 20 years after the American registry (the National Familial Pancreatic Tumor Registry, NFPTR) [30]. The significance of FPC remains to be undefined in a large extent in Asian counties. Establishing a screening program for early diagnosis is crucial to improving the prognosis of this intractable cancer. However, screening of general populations is not feasible because of the relatively low incidence of PDAC. Therefore, identification and evaluation of a risk of PDAC in FPC kindred may lead to establishment of highly efficient screening and improving of prognosis of PDAC in Asian populations.

In conclusion, 7.3% (88/1197) of PDAC patients in our Japanese cohort fulfilled the criterion of FPC; there was at least a pair of FDRs with PDAC in the kindred. We identified deleterious heterozygous germline mutations in well-established familial cancer-associated genes, *BRCA2*, *PALB2*, *ATM* and *MLH1*, in 14.5% (8/54) of our FPC patients. Our findings indicate that a subset of Japanese FPC patients may be associated with deleterious mutations of classical FPC genes as evidenced in Western populations.

## MATERIALS AND METHODS

### Ethics

The experimental protocols were approved by the institutional review board at the National Cancer Center (2013-292) and Tokyo Women's Medical University (213C). Written informed consent was obtained from all patients. The methods were carried out in accordance with the approved guidelines.

### Patients and tissue samples

We reviewed the National Cancer Center Hospital database and the Tokyo Women's Medical University Hospital database of patients with pathologically proven PDAC including its variants, namely, adenosquamous carcinoma, mucinous noncystic carcinoma, and undifferentiated carcinoma, between 2002 and 2013 and 2006 and 2011, respectively. Three patients treated in Kyorin University Hospital were included in cases of Tokyo Women's Medical University Hospital as consulting cases. On examination of medical interview sheets and medical records, cases with insufficient data were excluded.

We identified patients who fulfilled the criterion of FPC in which at least a pair of FDRs with PDAC and compared the following clinicopathologic features between them and PDAC patients without a family history of PDAC: gender, age, tumor location, clinical stage at diagnosis, risk factors (smoking history, diabetes mellitus and chronic pancreatitis), personal medical history of any cancer, and family histories of any cancer in FDRs (Table 1). Clinical stage was categorized following the UICC staging system (seventh edition).

### Massively parallel sequencing of target genes

Germline DNA was extracted from peripheral blood leukocytes. A custom capture kit was designed using NimbleDesign (NimbleGen, Madison, WI) targeting exons and splice sites of 21 genes known to be associated with hereditary predispositions for pancreatic, breast and ovarian cancers, namely, *ATM*, *BARD1*, *BRCA1*, *BRCA2*, *BRIP1*, *CDH1*, *CHEK2*, *MLH1*, *MRE11*, *MSH2*, *MSH6*, *MUTYH*, *NBN*, *PALB2*, *PMS1*, *PMS2*, *PTEN*, *RAD50*, *RAD51C*, *STK11*, and *TP53*. Libraries were created using the SeqCap EZ Library (NimbleGen) and KAPA Library Preparation Kits (Kapa Biosysytems, Wilmington, MA) according to the manufacturers' protocols. Massively parallel sequencing was performed on Illumina HiSeq2500 platforms (Illumina, San Diego, CA). Bases were called with the default setting using Illumina BCLFAST2 (Illumina). Paired-end reads were aligned to the human reference genome (GRCh37) using the Burrows-Wheeler Aligner [31]. A Genome Analysis Toolkit (GATK) was used to detect single-nucleotide substitutions and small insertions and deletions, using best practices from the GATK Website (available at: https://www.broadinstitute.org/gatk/) [32]. To maximize sensitivity to detect variants, no variant quality filters were applied. In-house script was applied to annotate variants.

### Variant characterization

Variants in 21 genes were considered for analysis if they were (1) called nonreference by GATK; (2) predicted to affect the protein sequence or the splice site (i.e., ±5 base pairs); and (3) had an allele frequency of less than 1% in the 1000 Genome project [33, 34], dbSNP [35], ExAC Browser (available at Exome Aggregation Consortium, http://exac.broadinstitute.org), or the Japanese genetic variation (available at the Human Genetic Variation Browser, http://www.genome.med.kyoto-u.ac.jp/SnpDB/).

Variants were classified according to ClinVar [36]. In addition, for *MLH1*, *MSH2*, *MSH6*, *MUTYH*, *PMS1*, and *PMS2*, classification was according to the InSiGHT consortium (available at: http://insight-group.org/variants/database/) [37]. For *BRCA1* and *BRCA2*, variants were classified using the database generated by Vallée et al. [38], accessed through the LOVD Website (available at: http://brca.iarc.fr/LOVD). These groups have classified large numbers of variants in *BRCA1*, *BRCA2*, *MLH1*, *MSH2*, *MSH6*, and *PMS2*, according to the International Agency for Research on Cancer system based on available information from the literature. Classes 1 and 2 are considered benign, class 3 is considered VUS, and classes 4 and 5 are considered pathogenic [39].

Rare non-synonymous variants that were not in these databases were classified based on the predicted effect on the protein product. Nonsense variants and variants changing the canonical splice-sites (i.e., ±2 base pairs), and frameshift insertions and deletions were considered pathogenic unless they occurred in the last exon. PROVEN (available at: http://provean.jcvi.org/index.php) [40], SIFT (available at: http://sift.jcvi.org) [41] and PolyPhen-2 (available at: http://genetics.bwh.harvard.edu/pph2/) [42] were used for identification of functional missense mutations along with a literature review. Taken together with this information, rare non-synonymous variants were classified as either deleterious, benign, or VUS.

### Sanger sequencing

Variants were validated by Sanger sequencing. Polymerase chain reaction amplification was carried out using 20 ng of DNA with intronic primers flanking targeted exons as previously reported [43]. Polymerase chain reaction products were sequenced by use of a M13F primer (5′-GTAAAACGACGGCCAGT-3′) or a M13R primer (5′-CAGGAAACAGCTATGACC-3′) incorporated into the forward and reverse primers of each primer pair, respectively. Sequencing data were analyzed with Sequencher 5.0.1 software (Gene Codes, Ann Arbor, MI).

### Statistics

Differences in variables between PDAC patients with and without a family history of PDAC were analyzed using χ^2^ test. *P* < 0.05 was considered statistically significant. All statistical analyses were performed using JMP ver.11 (SAS Institute, Cary, NC).

## SUPPLEMENTARY MATERIALS TABLE




