# Supplementary material for: Germline mutations in Japanese familial pancreatic cancer patients
Source: Oncotarget. 2016 Oct 6;7(45):74227–35. doi: 10.18632/oncotarget.12490 (PMC5342048; doi:10.18632/oncotarget.12490)
Supplement: Supplementary file 1 [file oncotarget-07-74227-s001.pdf]

Germline mutations in Japanese familial pancreatic cancer patients

SUPPLEMENTARY TABLE

Supplementary Table S1: Rare non-synonymous variants in 21 genes associated with hereditary predisposition for pancreatic, breast and ovarian cancers

See Supplementary File1
