# Supplementary material for: Germline mutations in Japanese familial pancreatic cancer patients
Source: Oncotarget. 2016 Oct 6;7(45):74227–35. doi: 10.18632/oncotarget.12490 (PMC5342048; doi:10.18632/oncotarget.12490)
Supplement: Supplementary file 2 [file oncotarget-07-74227-s002.docx]

| **Supplementary Table 1.** Rare non-synonymous variants in 21 genes associated with hereditary predisposition for pancreatic, breast and ovarian cancers. | | | | | | | | | | | | | | | | | | |
| --- | --- | --- | --- | --- | --- | --- | --- | --- | --- | --- | --- | --- | --- | --- | --- | --- | --- | --- |
|  | **Patient ID** | **Gender** | **Gene** | **Sub region** | **Type of mutation** | **Nucleotide change** | **Amino acid change** | **1000_ GenomeMAF (JPN)** | **ExAC Browser (East Asian)** | **HGVB SnpDB MAF** | **PROVEN (Score)** | **SIFT (Score)** | **Polyphen2 HDIV** | **Polyphen2HDIV_class** | **InSIGHT** | **LOVD IARC** | **ClinVar** | **Interpretation** |
| 1 | NCCH-9 | Male | *ATM* | CDS57 | Missense | c. 8581 A>G | p. Ile2861 Val | NA | NA | NA | Neutral (-0.63) | Damaging (0) | 0.999 | PROBABLY DAMAGING |  |  | NA | VUS |
| 2 | NCCH- 21 | Male | *NBN* | CDS5 | Missense | c.511 A>G | p. Ile171 Val | 0.005 | 0.002208 | 0.007908612 | Neutral (-0.8) | Damaging (0) | 1.000 | PROBABLY DAMAGING |  |  | Conflicting interpretations of pathogenicity | VUS |
| 3 | NCCH- 23 | Female | *BRCA1* | CDS9 | Missense | c. 3157 G>A | p. Glu1053 Lys | NA | NA | NA | NA | NA | 1.000 | PROBABLY DAMAGING |  | NA | NA | VUS |
| 4 | NCCH- 24 | Female | *MUTYH* | CDS4 | Missense | c. 377 G>A | p. Arg126 Gln | NA | NA | NA | Deleterious (-3.2) | Damaging (0.005) | NA | - | NA |  | Uncertain significance | VUS |
| 5 | NCCH- 34 | Male | *RAD50* | CDS25 | Inframe | c. 3811_3813 delGAA | p. Glu1271 del | NA | 0.0002312 | NA | Deleterious (-11.44) | NA | NA | - |  |  | NA | VUS |
| 6 | TWMU-3-1 | Male | *BRCA1* | CDS3 | Missense | c. 154 C>T | p. Leu52Phe | NA | 0.001752 | 0.004103967 | Neutral (-0.62) | Damaging (0) | 1.000 | PROBABLY DAMAGING |  | NA | Uncertain significance | VUS |
| VUS, variants of uncertain significance | | | | | | | | | | | | | | | | | | |
